# PD-L2 act as an independent immune checkpoint in colorectal cancer beyond PD-L1

**DOI:** 10.3389/fimmu.2024.1486888

**Published:** 2024-12-02

**Authors:** Lvyun Zhu, Ying Qu, Junru Yang, Tong Shao, Jingyu Kuang, Chuanyang Liu, Yanhua Qi, Ming Li, Yingying Li, Sujuan Zhang, Jingyang Wang, Yu Liu, Jiali Liu, Yanming Hu, Lingyun Zhu, Tao Hou

**Affiliations:** ^1^ Department of Biology and Chemistry, College of Sciences, National University of Defense Technology, Changsha, Hunan, China; ^2^ Laboratory of Liquid Propellant Application Technology, Jiuquan Satellite Launch Centre, Jiuquan, China; ^3^ Department of Oncology, The Second Xiangya Hospital, Central South University, Changsha, Hunan, China

**Keywords:** immune checkpoint blockade, colorectal cancer, antitumor immunity, therapeutic target, tumor microenvironment

## Abstract

**Introduction:**

Immunotherapy, especially immune checkpoint blockade (ICB), holds promise as a therapeutic strategy in colorectal cancer (CRC) by harnessing the patient’s immune system to target malignant cells. Particularly, the PD-1/PD-L1 axis is widely recognized for its critical role in tumor microenvironment immunosuppression. Antibodies targeting PD-1 or PD-L1 have shown sustained efficacy against various cancers, including CRC. Nonetheless, many CRC patients exhibit limited responses to such immunotherapy, and the resistance mechanisms remain incompletely understood.

**Methods:**

We conducted experiments with C57BL/6 mice, and used the MC38 cell line for ICB treatment studies in syngeneic mouse models. Gene and protein analyses were performed using qPCR, Western Blot, and flow cytometry, with bioinformatics for clinical data survival analysis.

**Results:**

In this study, we reveal that targeting PD-L2 emerges as a complementary therapeutic strategy to PD-1/PD-L1 blockade in CRC. Although PD-L2 is also inducible by IFNγ, like PD-L1, it displays a unique spatial distribution within the tumor microenvironment, implying discrete roles in immune evasion. Additionally, we uncovered a significant correlation between PD-L1 and PD-L2 expression levels and the infiltration of various immune cells, encompassing multiple dendritic cell (DC) subtypes. This correlation implies an enhanced antigen presentation process that may be unleashed by blocking these two immune checkpoints.

**Discussion:**

Our results highlight the significance of PD-L2 as an essential immune checkpoint alongside PD-L1 and emphasize its potential as a target for bolstering antitumor immunity in colorectal cancer.

## 1 Introduction

Colorectal cancer (CRC) ranks as the third most common cancer and the second leading cause of cancer-related mortality worldwide (1). Over the past decade, significant advancements have been made in understanding the immune microenvironment of CRC, leading to the development of novel immunotherapeutic strategies aimed at enhancing antitumor immunity. The most notable breakthroughs have come from the use of immune checkpoint inhibitors, particularly those targeting the programmed cell death protein 1 (PD-1)/programmed death-ligand 1 (PD-L1) axis (2, 3). Monoclonal antibodies against PD-1 (nivolumab and pembrolizumab) and PD-L1 (atezolizumab and durvalumab) have shown promising results, especially in patients with microsatellite instability-high (MSI-H) or mismatch repair-deficient (dMMR) tumors (4). These subtypes of CRC exhibit high mutational burdens, which correlate with increased neoantigen formation and enhanced response to immune checkpoint blockade (ICB) therapy. In the KEYNOTE-164 and KEYNOTE-177 trials, pembrolizumab demonstrated durable clinical benefit in MSI-H/dMMR metastatic CRC, leading to its approval by regulatory agencies for this specific patient population (5, 6).

Despite these successes, the majority of CRC patients do not benefit from monotherapy with checkpoint inhibitors due to the inherent immunosuppressive nature of the tumor microenvironment (7, 8). To overcome resistance mechanisms, combination therapies are being explored. Strategies include combining checkpoint inhibitors with chemotherapy, targeted therapies, and other immunomodulatory agents. For instance, the combination of pembrolizumab and chemotherapy has shown improved outcomes compared to chemotherapy alone in the first-line treatment of MSI-H/dMMR metastatic CRC, as evidenced in the KEYNOTE-176 trial (9). However, more efforts remain essential for the identification of predictive biomarkers for response and the elucidation of mechanisms of resistance in CRC immunotherapy, thereby paving the way for more personalized approaches and novel therapeutic strategies.

Apart from the PD-1/PD-L1 axis, which acts as pivotal immune checkpoints in the cancer immune microenvironment, programmed cell death ligand 2 (PD-L2), also known as B7-DC or CD273, is another ligand of PD-1. The interaction of PD-L2 with PD-1 results in an inhibitory signal that suppresses immune responses. Initially, PD-L2 expression was thought to be restricted to antigen-presenting cells such as dendritic cells and macrophages (10). However, recent studies have demonstrated that PD-L2 is also highly expressed in various human cancers, including head and neck squamous cell carcinoma, lung squamous cell carcinoma, renal cell carcinoma, pancreatic ductal adenocarcinoma, and cervical cancer (11–14). PD-L2 expression may correlate with specific clinical-pathological features and patient outcomes. For example, research has shown that PD-L2 expression levels might be associated with tumor stage, differentiation grade, overall survival and therapeutic efficacy (15, 16). A compelling study has shed light on the role of PD-L2 in CRC, revealing that overexpression of PD-L2 is significantly associated with inferior patient survival rates. This overexpression is hypothesized to be triggered by interferon-gamma (IFNγ) stimulation and heightened glycosylation modification (17). Although further validation across larger cohorts is needed, these findings underscore the importance of considering both PD-L1 and PD-L2 status in predicting the efficacy of anti-PD-1 immunotherapy. Moreover, despite the structural similarity between PD-L2 and PD-L1, the binding affinity between PD-L2 and PD-1 is two- to sixfold higher than that between PD-L1 and PD-1 (18). This suggests that PD-L2 plays a significant role in immune escape, as the strong interaction inhibits cytokine secretion and T-cell proliferation. Beyond its role in immune modulation, PD-L2 has also been shown to have a robust biological role during tumorigenesis, such as promoting invasion and triggering chemoresistance in human cancers (19). Given the importance of the PD-L2/PD-1 axis in cancer, there is growing interest in developing therapeutic strategies that target this checkpoint (20). However, the knowledge about the PD-L2 regulatory network is relatively limited, and there are currently no clinical practices or trials involving immunotherapy regimens specifically targeting PD-L2 in CRC. This highlights the need for in-depth research to reveal the role of PD-L2 in evading antitumor immunity in CRC and to elucidate its functional relationship with PD-L1 within the tumor microenvironment.

In this study, we have delineated the role of PD-L2 as a pivotal immune checkpoint in CRC, acting in concert with PD-L1. Our observations suggested that PD-L2 could serve as a significant biomarker for patient stratification in ICB therapy and represents a promising therapeutic target when combined with existing immune checkpoints.

## 2 Materials and methods

### 2.1 Animals

Six- to eight-week-old both sexes C57BL/6 mice were sourced from commercial vendors and used as transplant hosts. All animals were maintained in standard individually ventilated, pathogen-free conditions, with a light-dark cycle of 12 h and temperature around 21-25°C, humidity of 40–60%. All animals’ experiments follow ARRIVE guidelines and followed strict randomization.

### 2.2 Cell lines

MC38 cell line was obtained from the National Infrastructure of Cell Line Resource (Beijing, China) and authenticated by STR profiling. The MC38 cell line, stably expressing TagRFP657, was engineered through a lentivirus-mediated gene transfer approach. The Lenti-TagRFP657 expression plasmid, which was utilized in this construction, was sourced from the Addgene repository (Plasmid #212961) (21). Cells were cultured in Dulbecco’s Modified Eagle Medium (DMEM) supplemented with 10% Fetal Bovine Serum (FBS), 1% Penicillin-Streptomycin (Penstrep), and maintained in a humidified CO_2_ incubator set to 5% CO_2_ at 37°C. Regular testing using a Mycoplasma Detection Kit (Thermo Fisher Scientific) or conventional PCR ensured that all cell lines were free of Mycoplasma contamination. To mitigate the risk of phenotypic drift, cells were cryopreserved in multiple aliquots upon receipt.

### 2.3 Clinical samples

Colorectal cancer tissue samples were collected during surgical procedures at the Second Xiangya Hospital, Central South University, Changsha, China. All individuals underwent primary tumor resection and provided informed consent for the use of their tissue in research. The study adhered strictly to ethical guidelines for human subject research, and the protocols were approved by the Central South University Ethics Committee (Approval No. Z0449-01).

### 2.4 Syngeneic mouse model with ICB treatment

MC38 cells were inoculated subcutaneously into the right rear groin of C57BL/6 mice at a density of 2 × 10^6^ cells per site. Tumor growth was meticulously monitored, with size measurements recorded every 3 days using vernier calipers. Mice were randomly assigned into 5 groups to receive treatment of PBS, 8 mg/kg anti-PD-1, 8 mg/kg anti-PD-L1, 8 mg/kg anti-PD-L2 or a combination with 4 mg/kg anti-PD-L1 and 4 mg/kg anti-PD-L2, for 3 doses at the indicated times.

### 2.5 Orthotopic mouse model with ICB treatment

Male C57BL/6 mice, 6-8 weeks old, were anesthetized, and a small incision was made to expose the intestine. Under microscopic visualization, a 50 µl suspension containing 2 × 10^6^ cells was injected into the intestine wall. The intestine was then returned to the peritoneal cavity, and the incision was closed with sutures. Mice were monitored for recovery and provided with appropriate analgesics. They were observed for any signs of distress or complications. Tumor growth were assessed at the end of the study period, typically 15 days post-implantation, by euthanizing the mice and performing necropsies to examine tumor progression.

### 2.6 Quantitative PCR assay

Total RNA was extracted from MC38 cells in response to different concentration of IFNγ using TRIzol reagent (Invitrogen) and subsequently reverse transcribed into complementary DNA (cDNA) with SuperScript III Reverse Transcriptase (Invitrogen), following the manufacturer’s protocol. Quantitative PCR (qPCR) was conducted in 96-well plates using the SYBR Green PCR Master Mix (Roche). The fluorescence intensity was measured using a Light Cycler 480 instrument (Roche). A forward primer of 5’-AGTCAATGCCCCATACCGC-3’ and a reverse primer of 5’-TTCTGGATAACCCTCGGCCT-3’ were used for mouse PD-L1 amplification. A forward primer of 5’-TCATTGACCCTCTGAGTCGG-3’ and a reverse primer of 5’-GGAAGATCAAAGCGATGGTGC-3’ were used for mouse PD-L2 amplification. A forward primer of 5’-GGCACCACACCTTCTACAATG-3’ and a reverse primer of 5’-GTGGTGGTGAAGCTGTAGCC-3’ were used for mouse β-actin amplification.

### 2.7 Western blot

Cells were lysed with the Cell Lysis Buffer (Cell Signaling). After incubation on ice for a minimum of 10 minutes, the lysates were analyzed for protein concentration using the bicinchoninic acid (BCA) assay. Subsequently, 20 μg of each protein sample was loaded and separated via SDS-PAGE, then transferred onto PVDF membranes (Merck Chemicals). Following membrane blocking, primary antibodies were applied overnight at the indicated concentrations in a 2% BSA-TBST solution: anti-PD-L1, anti-PD-L2, anti-β-actin (all from Abcam). Subsequently, the membranes were incubated with goat anti-rabbit HRP (ThermoFisher) for 1 hour. Detection was performed using the SuperSignal™ West Pico PLUS chemiluminescent substrate (ThermoFisher).

### 2.8 Multicolor immunohistochemical analysis

Paraffin-embedded tumor tissue sections were deparaffinized in xylene and subjected to a graded ethanol series for rehydration. Antigen retrieval was performed using heat in citrate buffer. The slides were then blocked with 5% goat serum in phosphate-buffered saline (PBS), and subsequently incubated with primary antibodies specific for PD-L1, PD-L2, MHC-II/HLA-DR and CD11c (AiFang biological). Nuclei were counterstained with DAPI. A TSA indirect kit (AiFang biological) was used according to the manufacturer’s instructions. Imaging was conducted using the Vectra^®^ Polaris™ Imaging System (Akoya Biosciences), and subsequent image analysis was performed utilizing the HALO™ Image Analysis Software.

### 2.9 Flow cytometry

For IFNγ stimulation experiment *in vitro*, single-cell suspensions were incubated with a rat anti-mouse CD16/CD32 monoclonal antibody (ThermoFisher) to block Fc receptor-mediated reactions, followed by the surface staining at 4°C for 15 min. Fluorescein conjugated antibodies including PE-anti-PD-L1, PE-anti-PD-L2, Alexa Fluor 700-PD-L1 and PerCP-Cy5.5-PD-L2 (BioLegend) were used for surface staining. For MC38 cell-derived mouse tumor analysis *in vivo*, tumor tissues were harvested, minced, and treated with a digestion solution comprising PBS with 2% FBS, collagenase type IV (0.25 mg/mL, Sigma), and DNase I (20 U/mL, Sigma). The mixture was then agitated on a shaker incubator at 37°C for 10 minutes to facilitate disaggregation, followed by vigorous pipetting with a 10 mL pipette. The resulting cell suspension was strained through a 70 μm nylon mesh to obtain a single-cell suspension. The single-cell suspensions were incubated with a rat anti-mouse CD16/CD32 monoclonal antibody (ThermoFisher) to block Fc receptor-mediated reactions, followed by the surface staining at 4°C for 15 min. A panel of antibodies (BioLegend) was used for surface staining: Alexa Fluor 700-PD-L1, PerCP-Cy5.5-PD-L2, and Zombie Violet Fixable Viability Kit. The samples were analyzed using a fluorescent-activated cell sorter (FACS) Canto II Cell Analyzer (BD Biosciences) with BD FACSDiva Software (v9.0, BD Biosciences). Data analysis was conducted using FlowJo software (Ashland).

### 2.10 Bioinformatic analysis of clinical data

Clinical gene expression and survival data (including overall survival, n = 976 and progression-free survival, n = 571) were obtained from the immunotherapy section of the KM-plotter platform (22). Survival analysis was performed using the R programming language to perform survival analysis based on the Kaplan-Meier method to compare survival probabilities between the two treatment groups. Statistical significance was assessed using the log-rank test, with a p-value of less than 0.05 deemed significant for differences between groups. Additionally, the hazard ratio (HR) with 95% confidence intervals was calculated to quantify the relative risk associated with each treatment modality (including anti-PD-1 treatment and anti-PD-L1 treatment). The optimal cut-off value was determined using the surv_cutpoint function from the survminer package in R, and the survival curves were visualized using the ggsurvplot function.

### 2.11 Statistics and reproducibility

Data between two groups were analyzed using a two-tailed unpaired t-test. Multiple t-test using the Holm-Sidak method was used for multiple group comparison. Different levels of statistical significance were accessed based on specific p values and type I error cutoffs (0.05 and 0.01). GraphPad Prism Software and RStudio were used for these analyses.

## 3 Results

### 3.1 Combined PD-L1/PD-L2 blockade essential for halting CRC progression

**Figure 1:**
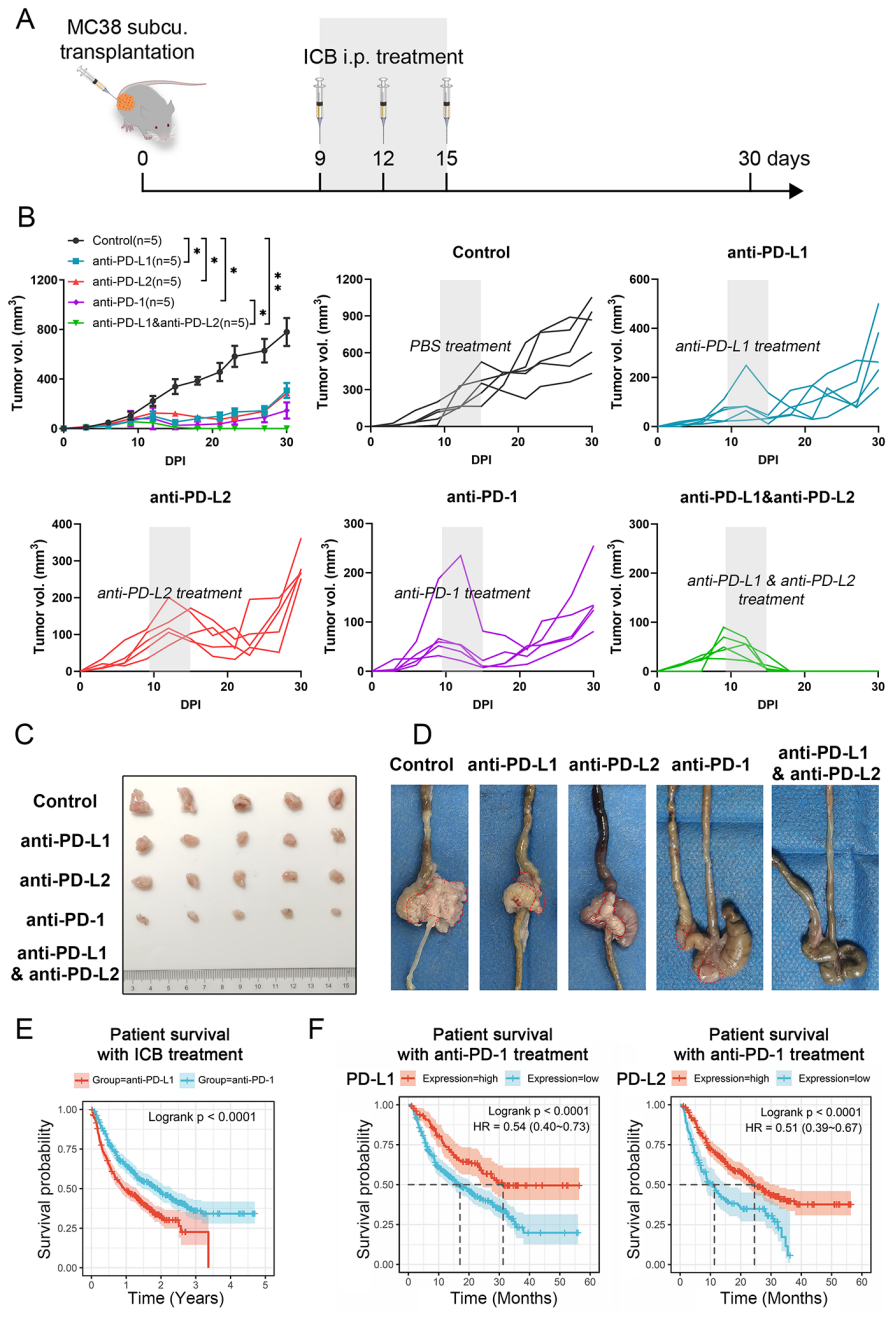
Impact of different ICB therapies on CRC progression. **(A)** Schematic of the experimental design. **(B)** Primary tumor growth curve of C57BL/6 mice bearing MC38 cell-derived tumors, treated with either PBS control, anti-PD-L1, anti-PD-L2, anti-PD-1 or a combination of anti-PD-L1 and anti-PD-L2. **(C)** Representative photographs of tumors from subcutaneous tumor transplant at 30 DPI. **(D)** Representative photographs of tumors with orthotopic tumor transplant at 15 DPI. Tumors were circled out by dashed lines. **(E)** Kaplan-Meier survival curves for overall survival of patients undergoing anti-PD-L1 or anti-PD-L2 therapies. **(F)** Kaplan-Meier survival analysis correlating PD-L1 or PD-L2 expression levels with overall survival in CRC patients treated with anti-PD-1. Error bars: All data points in this figure are presented as mean ± SE. Two-tailed unpaired t test. *, *P* < 0.05; **, *P* < 0.01.

To clarify the roles of PD-L1 and PD-L2 as immune checkpoints in CRC, we assessed the effects of their respective inhibitors, along with a PD-1 inhibitor, on tumor growth *in vivo*. Using the MC38 cell line, a representative of MSI-H CRC subtype, we established a syngeneic C57/BL6 murine model that faithfully replicates the immune landscape. Mice were subcutaneously implanted with MC38 cells in the right groin flank, and based on initial tumor size measurements, were evenly divided into three cohorts for treatment with either PBS control, anti-PD-L1, anti-PD-L2, anti-PD-1 or a combination of anti-PD-L1 and anti-PD-L2 (**Figure 1A**). Monotherapies with anti-PD-L1, anti-PD-L2, or anti-PD-1 substantially decelerated the progression of CRC. However, they did not achieve complete tumor eradication, as regrowth was observed approximately 24 days post injection (DPI) of tumor cells. Notably, anti-PD-1 therapy demonstrated superior efficacy in tumor rejection compared to anti-PD-L1 and anti-PD-L2 therapies. In sharp contrast, the combinatorial therapy targeting both PD-L1 and PD-L2 led to complete tumor regression (**Figures 1B, C**). Similar results could also be observed in orthotopic transplantation models (**Figure 1D**). Analysis of clinical data from The Cancer Genome Atlas (TCGA) corroborated these findings, showing that CRC patients treated with anti-PD-1 had improved overall survival compared to those treated with anti-PD-L1 (**Figure 1E**). Notably, patients with high PD-L1 or PD-L2 expression levels demonstrated significant survival advantages with anti-PD-1 therapy; however, the survival benefit was more pronounced in those with elevated PD-L1 levels compared to PD-L2 (**Figure 1F**). These results underscore the synergistic role of PD-L1 and PD-L2 as immune checkpoints, highlighting the necessity of their combined inhibition for effective colorectal tumor regression.

### 3.2 Stimulated PD-L1 and PD-L2 expression in response to IFNγ in CRC cells

**Figure 2:**
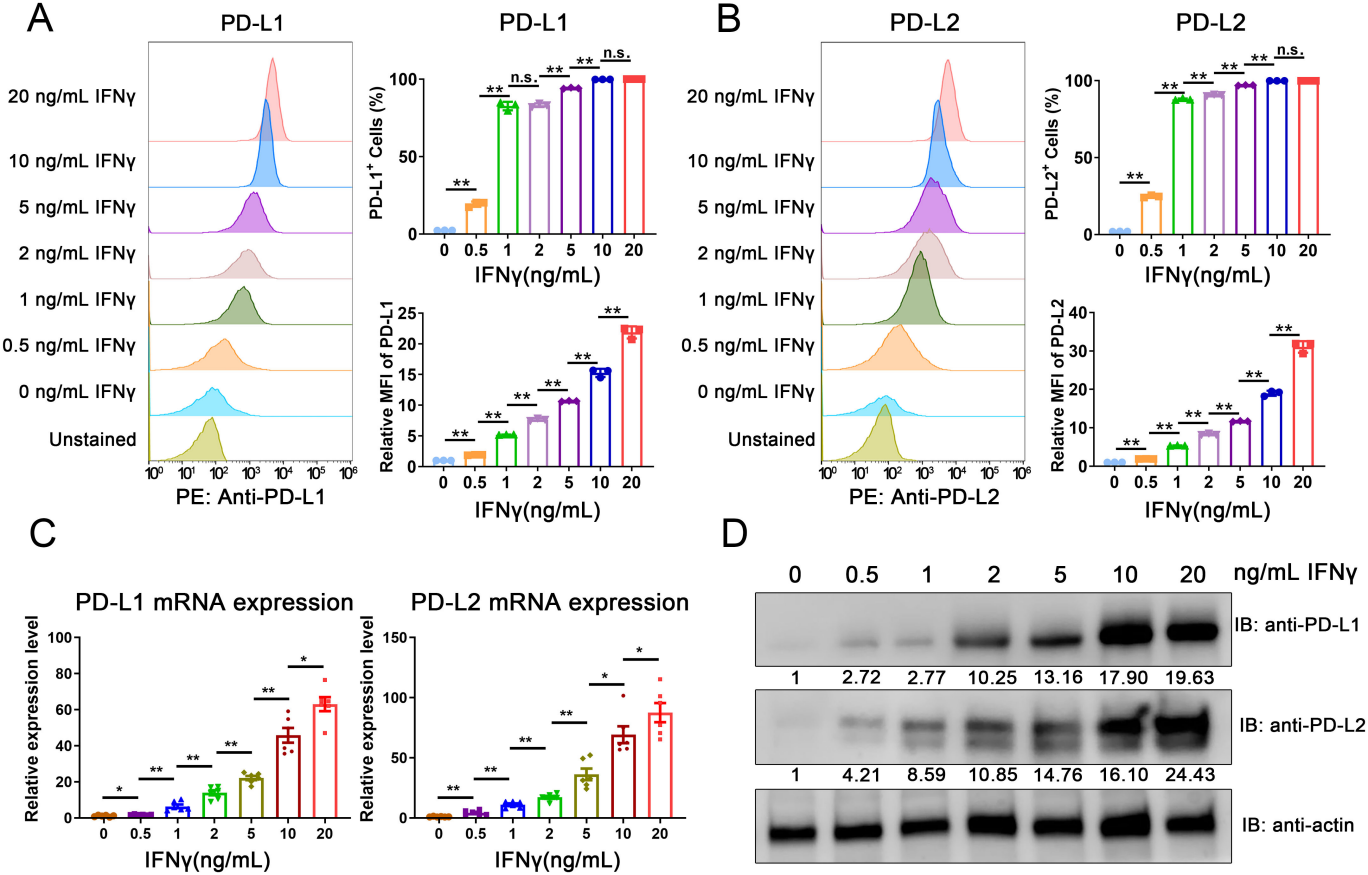
IFNγ-mediated induction of PD-L1 and PD-L2 in CRC cells. **(A, B)** Flow cytometric analysis of surface expression levels of PD-L1 **(A)** and PD-L2 **(B)** in the MC38 mouse CRC cell line following IFNγ treatment. **(C)** qPCR analysis depicting the induction of PD-L1 and PD-L2 mRNA expression in MC38 cells in response to IFNγ. **(D)** Western blot analysis confirming the increased expression of PD-L1 and PD-L2 proteins in MC38 cells upon IFNγ exposure. The relative grayscale value of bands probed with anti-PD-L1 and anti-PD-L2 antibodies was normalized to β-actin levels within each sample set. Data from the PBS-treated control group are normalized to a value of 1. Error Bars: Presented as mean ± standard error of the mean (SEM) for all data points in this figure. Statistical significance was determined using a two-tailed unpaired t-test. n.s., not significant; *, *P* < 0.05; **, *P* < 0.01.

To assess the regulatory effects of IFNγ on PD-L1 and PD-L2, we exposed the MC38 mouse CRC cell line to varying concentrations of IFNγ. Flow cytometry revealed a dose-dependent increase in the cell surface expression of both PD-L1 and PD-L2 following IFNγ exposure (**Figures 2A, B** and **Supplementary Figure S1**). Consistent with these findings, the induction of PD-L1 and PD-L2 at both the mRNA and protein levels was confirmed through quantitative PCR (qPCR) and Western blot analysis, respectively (**Figures 2C, D**). These data imply that the upregulation of PD-L1 and PD-L2 on tumor cells in response to immune cell-secreted IFNγ may represent a critical immune evasion strategy in CRC.

### 3.3 Independent action of PD-L1 and PD-L2 as immune checkpoints in CRC progression

**Figure 3:**
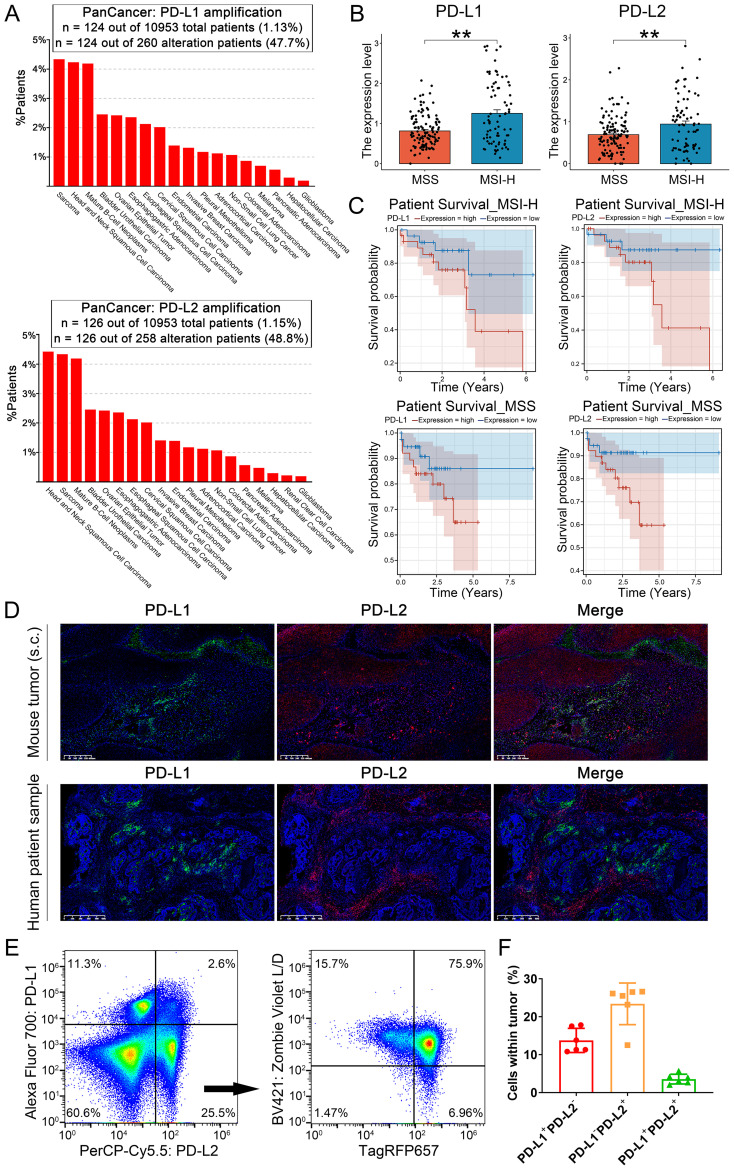
Role of PD-L1 and PD-L2 as immune checkpoints in CRC progression. **(A)** Bar plot illustrating the proportion of patients with PD-L1 and PD-L2 amplification mutations among various cancer types. **(B)** Expression of PD-L1 and PD-L2 among CRC patients, stratified by microsatellite instability status. **(C)** Univariable Cox regression curves for overall survival of CRC patients, stratified by PD-L1 and PD-L2 expression levels and microsatellite instability status. **(D)** Multicolor immunohistochemical analysis of PD-L1 and PD-L2 on tissue sections from MC38 cell-derived mouse tumors and human CRC biopsies TSA approach. Green: TYR520-anti-PD-L1; Red: TYR570-anti-PD-L2; Blue: DAPI. **(E)** Flow cytometric analysis of PD-L1 and PD-L2 expression on mouse tumors derived from MC38 cells with stable TagRFP657 expression. The right chart depicting the proportion of TagRFP657^+^ tumor cells within PD-L2^+^PD-L1^-^ cells. **(F)** Statistic analysis of PD-L1^+^PD-L2^-^, PD-L1^-^PD-L2^+^ and PD-L1^+^PD-L2^+^ cells in tumors as identified by flow cytometry. Data were analyzed from 6 independent samples. Error Bars: Presented as mean ± SEM for all data points in this figure. **, *P* < 0.01.

To explore the synergistic influence of PD-L1 and PD-L2 on CRC prognosis, we analyzed clinical data from the TCGA database. Our examination revealed that both PD-L1 and PD-L2 are frequently amplified in various human cancers, with an overall amplification mutation rate of 1-4% among cancer patients (**Figure 3A**). The expression analysis revealed that patients with MSI-H CRC exhibit significantly elevated expression of both PD-L1 and PD-L2 compared to their MSS counterparts, highlighting a distinct immunological profile associated with the MSI-H subtype (**Figure 3B**). Survival analysis demonstrated a significant correlation between high expression levels of PD-L1 and PD-L2 and reduced survival outcomes for both MSI-H and MSS CRC patients (**Figure 3C** and **Supplementary Figure S2**). To elucidate the spatial relationships of PD-L1 and PD-L2 within the tumor microenvironment, we conducted multicolor immunohistochemical analysis on tissue sections from both MSI-H MC38 cell-derived mouse tumors and human CRC samples using tyramide signal amplification (TSA) approach. Obviously, PD-L1 and PD-L2 exhibited distinct patterns of distribution across tumor tissues (**Figure 3D**). This observation was corroborated by flow cytometry, which seldom detected co-expression of PD-L1 and PD-L2 on tumor cells (**Figures 3E, F**). In the case of tumors derived from MC38 cells with stable TagRFP657 expression, our analysis revealed that the majority of PD-L2^+^ cells in mouse tumors were also TagRFP657^+^, suggesting that PD-L2 is predominantly expressed on tumor cells (**Figure 3E**). Collectively, these findings imply that each immune checkpoint molecule plays a distinct and independent role in the progression of CRC.

### 3.4 PD-L1 and PD-L2 expression linked to dendritic cell infiltration in CRC

**Figure 4:**
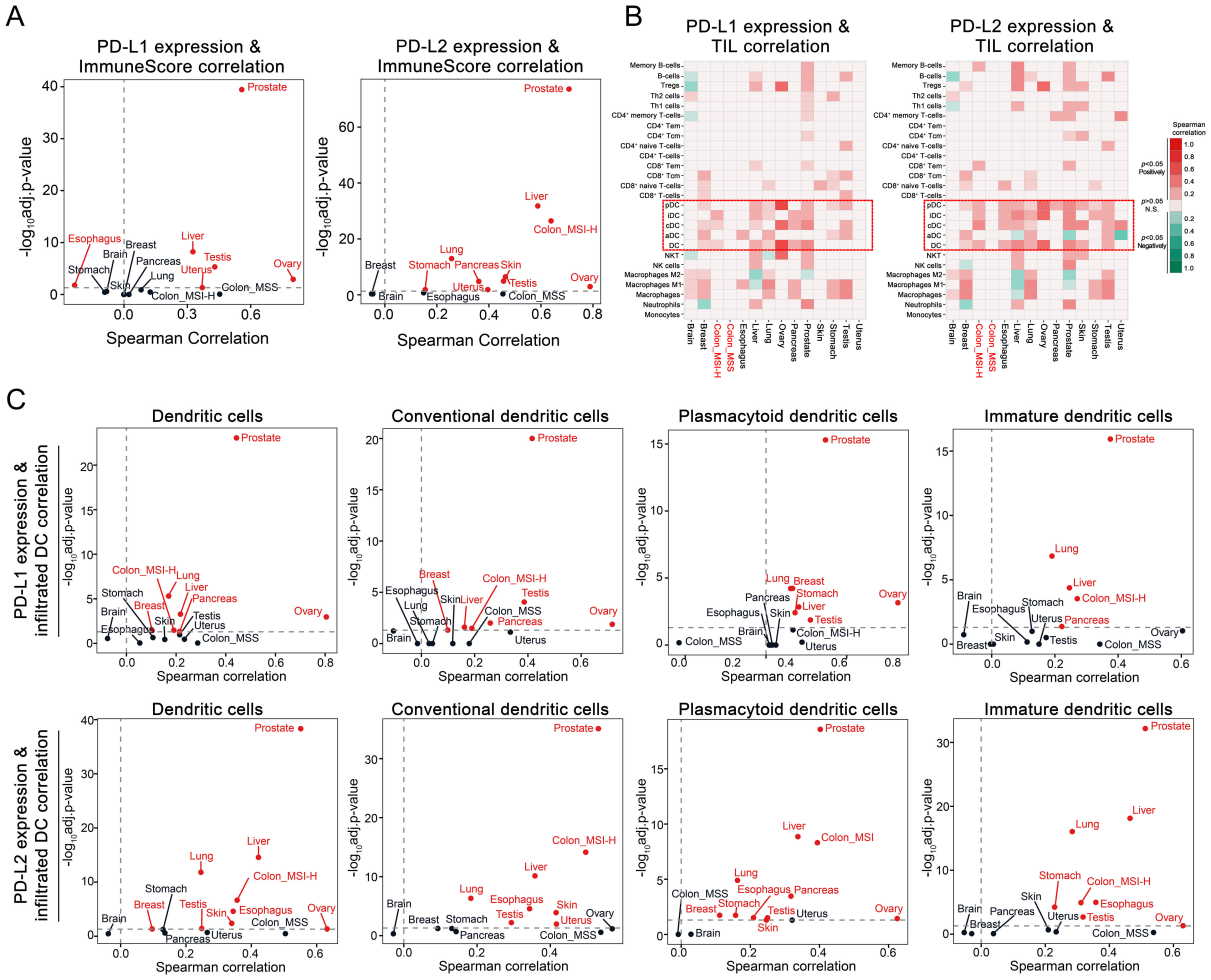
Correlation of PD-L1 and PD-L2 expression with tumor immune infiltration in CRC. **(A)** Volcano plot depicting the Spearman correlation coefficients for PD-L1 and PD-L2 mRNA levels with the immune score as a measure of global immune infiltration across diverse cancer types. Cancer types with significant positive correlations are marked in red (adjusted *P* < 0.05). **(B)** Heatmap illustrating the Spearman correlation between PD-L1 and PD-L2 mRNA levels and the infiltration of various immune cell types within tumors, with DC and its subsets highlighted by dashed boxes. **(C)** Volcano plot depicting the Spearman correlation coefficients for PD-L1 and PD-L2 mRNA levels with infiltration of DC and its subsets. Cancer types with significant positive correlations are marked in red (adjusted *P* < 0.05).

**Figure 5:**
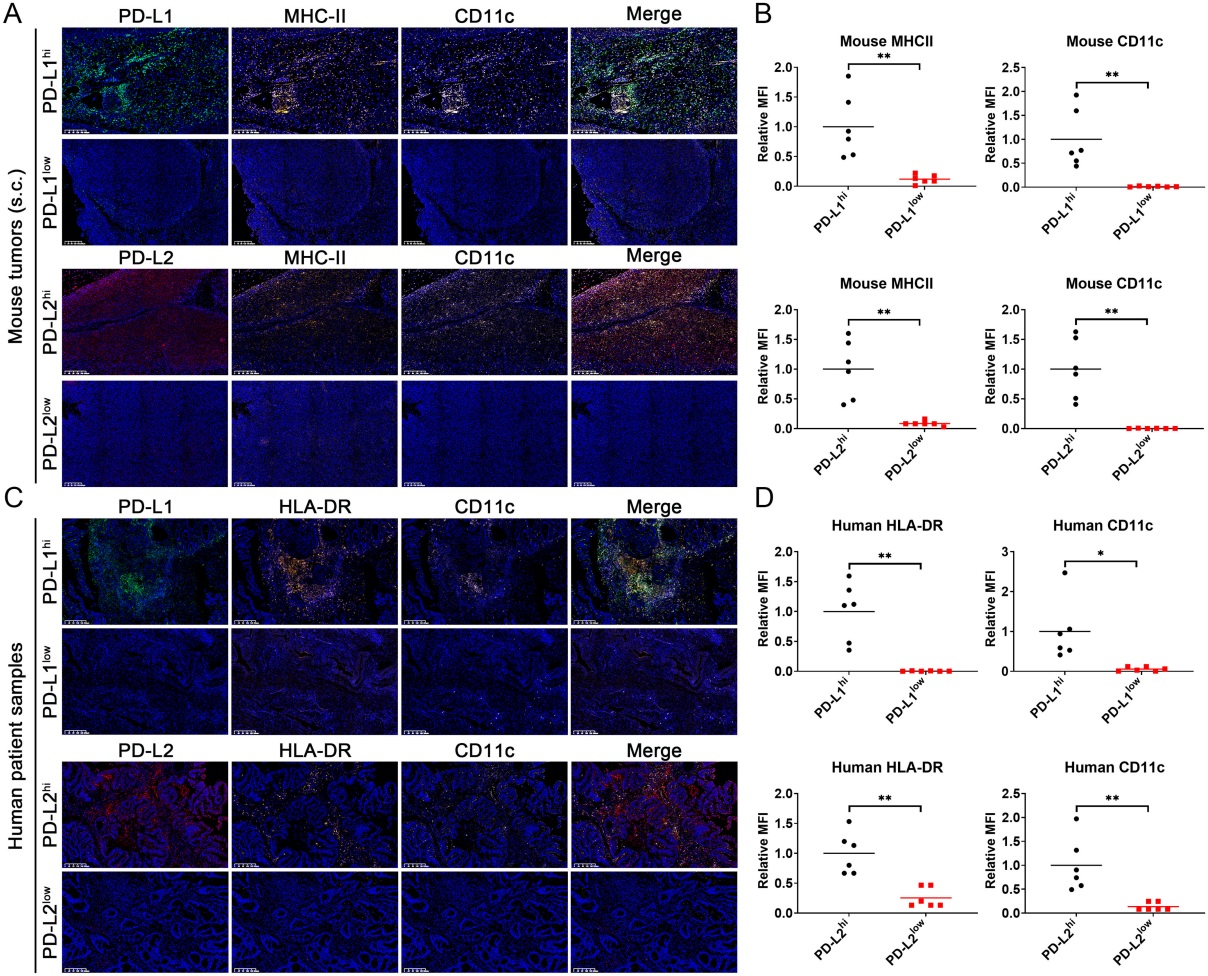
Multicolor immunohistochemical analysis of PD-L1, PD-L2, MHC-II/HLA-DR and CD11c on tissue sections from MC38 cell-derived mouse tumors and human CRC biopsies using TSA approach. **(A)** Staining image of mouse tumor sections. **(B)** Statistic analysis of relative mean fluorescence intensity (MFI) in the images staining for mouse MHC-II and CD11c. **(C)** Staining image of human tumor sections. **(D)** Statistic analysis of relative mean fluorescence intensity (MFI) in the images staining for mouse HLA-DR and CD11c. Green: TYR520-anti-PD-L1; Red: TYR570-anti-PD-L2; Orange: TYR620-anti-MHC-II/HLA-DR; Pink: TYR690-anti-CD11c; Blue: DAPI. Mean data from the PD-L1 or PD-L2 high-expression groups are normalized to a value of 1. Error Bars: Presented as mean ± standard error of the mean (SEM) for all data points in this figure. Statistical significance was determined using a two-tailed unpaired t-test. *, *P* < 0.05; **, *P* < 0.01.

To further investigate the impact of PD-L1 and PD-L2 expression on tumor immune microenvironment in CRC, we conducted the tumor immune infiltration analysis using both clinical datasets and tissue samples. Examination of TCGA data sets disclosed a significant positive correlation between PD-L1 and PD-L2 expression levels and the extent of overall immune cell infiltration across different cancer types, including the MSI-H CRC subtype (**Figure 4A**). Notably, expression of both PD-L1 and PD-L2 was positively associated with the infiltration of DCs, including conventional DCs, plasmacytoid DCs, and immature DCs, within MSI-H colorectal tumors. In contrast, this association was largely absent in MSS colorectal tumors, indicating a distinct immunological profile between MSI-H and MSS tumor subtypes (**Figures 4B, C**). Parallel findings were observed in tissue sections from MC38 cell-derived mouse tumors and human CRC biopsies. Multicolor immunohistochemical analysis showed a notable enrichment of MHC-II^+^CD11c^+^ cells, which are indicative of potential DCs, in tumor tissues exhibiting elevated levels of PD-L1 and PD-L2 (**Figure 5**). These findings suggest a possible compensatory mechanism of increased DCs infiltration in correlation to higher PD-L1 and PD-L2 expression, potentially underpinning the rationale for the effectiveness of combined PD-L1 and PD-L2 blockade therapies particularly, particularly in the treatment of MSI-H CRC patients.

## 4 Discussion

ICB therapy targeting the PD-1/PD-L1 axis has demonstrated remarkable success, offering clinical benefits across various tumor types, including chemoresistant and metastatic cancers (23). However, its effectiveness in solid tumors such as CRC has been limited, with many patients showing no response (24, 25). Notably, PD-L2, the alternative PD-1 ligand, has been less explored, partly due to its lower expression frequency in cancers compared to PD-L1 (26).

Recent clinical studies have identified PD-L2 expression as an independent predictor of response to PD-1 monoclonal antibody therapy in head and neck squamous cell carcinoma (11). Additionally, the natural compound zinc undecylenate, a small molecule inhibitor of PD-L2, has shown significant antitumor effects in cancers resistant to epidermal growth factor receptor-tyrosine kinase inhibitors (EGFR-TKIs) (15). Our study confirms that targeting PD-L2 is a promising therapeutic strategy that complements PD-1/PD-L1 blockade in CRC. We found that PD-L2, while similarly inducible by IFNγ as PD-L1, exhibits distinct spatial distribution within the tumor microenvironment, suggesting independent contributions to immune evasion. Furthermore, we discovered a strong association between PD-L1 and PD-L2 expression and immune cell infiltration such as various DC subtypes, indicating a potential mechanism by which ICB therapy may enhance antigen presentation within the tumor microenvironment.

An emerging question pertains to the coordination between PD-L2 and PD-L1 as immune checkpoints within the tumor microenvironment. Recent research has highlighted that PD-L1 and PD-L2, while paralogous genes with similar promoter regions, exhibit distinct regulatory mechanisms in tumor cells. Specifically, PD-L1 expression is predominantly governed by the IFNγ receptor signaling pathway, involving kinases JAK1 and JAK2, various STAT proteins, and other pathway modulators, culminating in the binding of IRF1 to the PD-L1 promoter. In contrast, PD-L2 expression is influenced by both IFNβ and IFNγ, with transcription factors STAT3 and IRF1 binding to its promoter region (27). Additionally, PD-L2 expression is uniquely susceptible to upregulation by interleukin-4 (IL-4), a distinction not shared by PD-L1 (28). Moreover, the repulsive guidance molecule B (RGMb), found on CD8^+^ T cells, serves as a distinct receptor for PD-L2, lacking an affinity for PD-L1. This interaction is crucial as it mediates the specific inhibitory effects of PD-L2 on CD8^+^ T cell (29). In this study, we elucidated the independent role of PD-L2 and PD-L1 in immune evasion, spatial distribution within tumors, and association with DCs infiltration in the tumor microenvironment. Such findings bolster the understanding of the coordinated mechanisms between PD-L2 and PD-L1 within the tumor microenvironment and reinforce the rationale for exploring PD-L2 as an alternative immunotherapy target.

In conclusion, our findings support the potential of combined PD-L1/PD-L2 blockade for superior therapeutic outcomes against CRC, especially for patients non-responsive to traditional ICB therapies. These insights may unlock the potential of PD-L2 as an immunotherapy target, expanding the reach of ICB therapy to a wider range of CRC patients. Given that the cell line and mouse model used in this study are a representative of MSI-H CRC subtype, it is imperative that future studies extend the focus to encompass microsatellite stable (MSS) tumor cells and orthotopic animal models. Moreover, unraveling the intricate interplay between PD-L1/PD-L2 expression and the broader immune cell infiltration through systematic approaches such as spatial transcriptomics will be a critical area of investigation in the future research. We eagerly anticipate the forthcoming research that will not only enhance our understanding of the intricate dialogue among immune checkpoints but also evaluate the clinical potential of these interactions as therapeutic targets.

## 5 Study Highlights

### 5.1 What is the current knowledge on the topic?

Immune checkpoint blockade therapies targeting PD-1 or PD-L1 have emerged as promising treatments for a spectrum of cancers. However, in the context of CRC, many patients show limited responses to these immunotherapies, with the underlying resistance mechanisms not yet fully elucidated. PD-L2, an alternative ligand for PD-1, is gaining recognition as a key player in immune evasion. Yet, current understanding of PD-L2’s role in mediating immune escape and its functional interplay with PD-L1 remains preliminary. To date, there are no clinical trials or practices focused on immunotherapy regimens that specifically target PD-L2 in CRC.

### 5.2 What question did this study address?

This study reveals that PD-L2, though similarly inducible by IFNγ as PD-L1, displays a distinct spatial distribution within the tumor microenvironment. This finding implies that PD-L2 may independently contribute to immune evasion. Furthermore, our research demonstrates a robust correlation between the expression levels of PD-L1 and PD-L2 and the infiltration of immune cells, including diverse DC subtypes. This correlation suggests a potential mechanism whereby ICB therapy could enhance antigen presentation within the tumor microenvironment.

### 5.3 What does this study add to our knowledge?

This study elucidated the independent role of PD-L2 and PD-L1 in immune evasion, their spatial distribution within tumors, and their association with DCs infiltration within the tumor microenvironment. These insights may advance our comprehension of the synergistic mechanisms between PD-L2 and PD-L1, underscoring the imperative to consider PD-L2 as a viable alternative target for immunotherapeutic interventions.

### 5.4 How might this change clinical pharmacology or translational science?

In light of these results, PD-L2 stands out as a promising target for ICB therapy, especially in combination with drugs that target established immune checkpoints.

## Data Availability

The original contributions presented in the study are included in the article/**Supplementary Materials**. Further inquiries can be directed to the corresponding author/s.

## Supplementary material

Supplementary Figure 1 Flow cytometric analysis of the co-expression of PD-L1 and PD-L2 on the MC38 cell line in response to various concentrations of IFNγ. Error Bars: Presented as mean ± standard error of the mean (SEM) for all data points in this figure. Statistical significance was determined using a two-tailed unpaired t-test. *, *P* < 0.05; **, *P* < 0.01.

Supplementary Figure 2 Univariable Cox regression curves for overall survival of CRC patients, stratified by PD-L1 and PD-L2 expression levels.
