# Supplementary figures and images for: PD-L2 act as an independent immune checkpoint in colorectal cancer beyond PD-L1
Source: Front Immunol. 2024 Dec 2;15:1486888. doi: 10.3389/fimmu.2024.1486888 (PMC11646888; doi:10.3389/fimmu.2024.1486888)

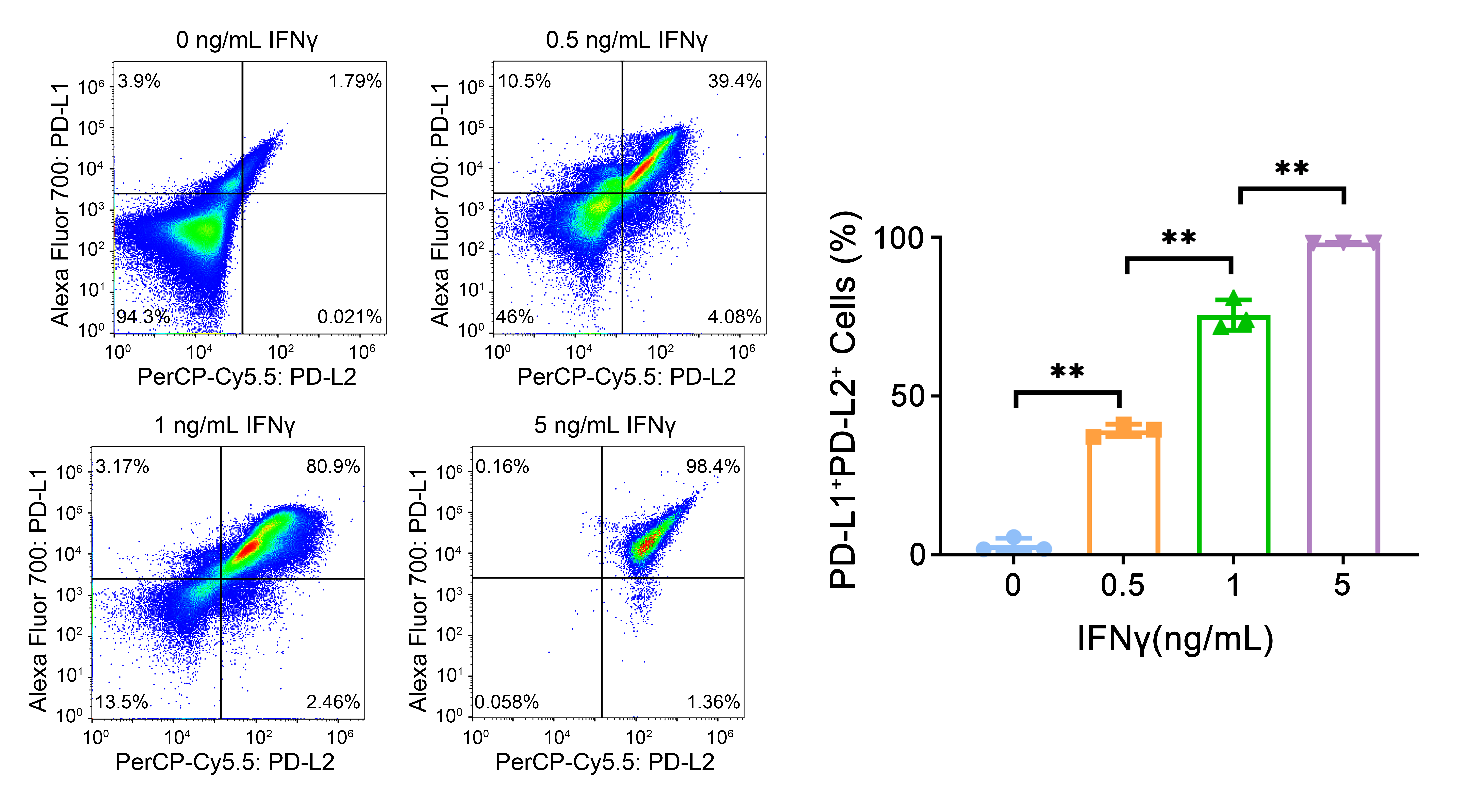

Supplement: Supplementary Figure 1 — Flow cytometric analysis of the co-expression of PD-L1 and PD-L2 on the MC38 cell line in response to various concentrations of IFNγ. Error Bars: Presented as mean ± standard error of the mean (SEM) for all data points in this figure. Statistical significance was determined using a two-tailed unpaired t-test. *, P < 0.05; **, P < 0.01. [file Image1.tif]

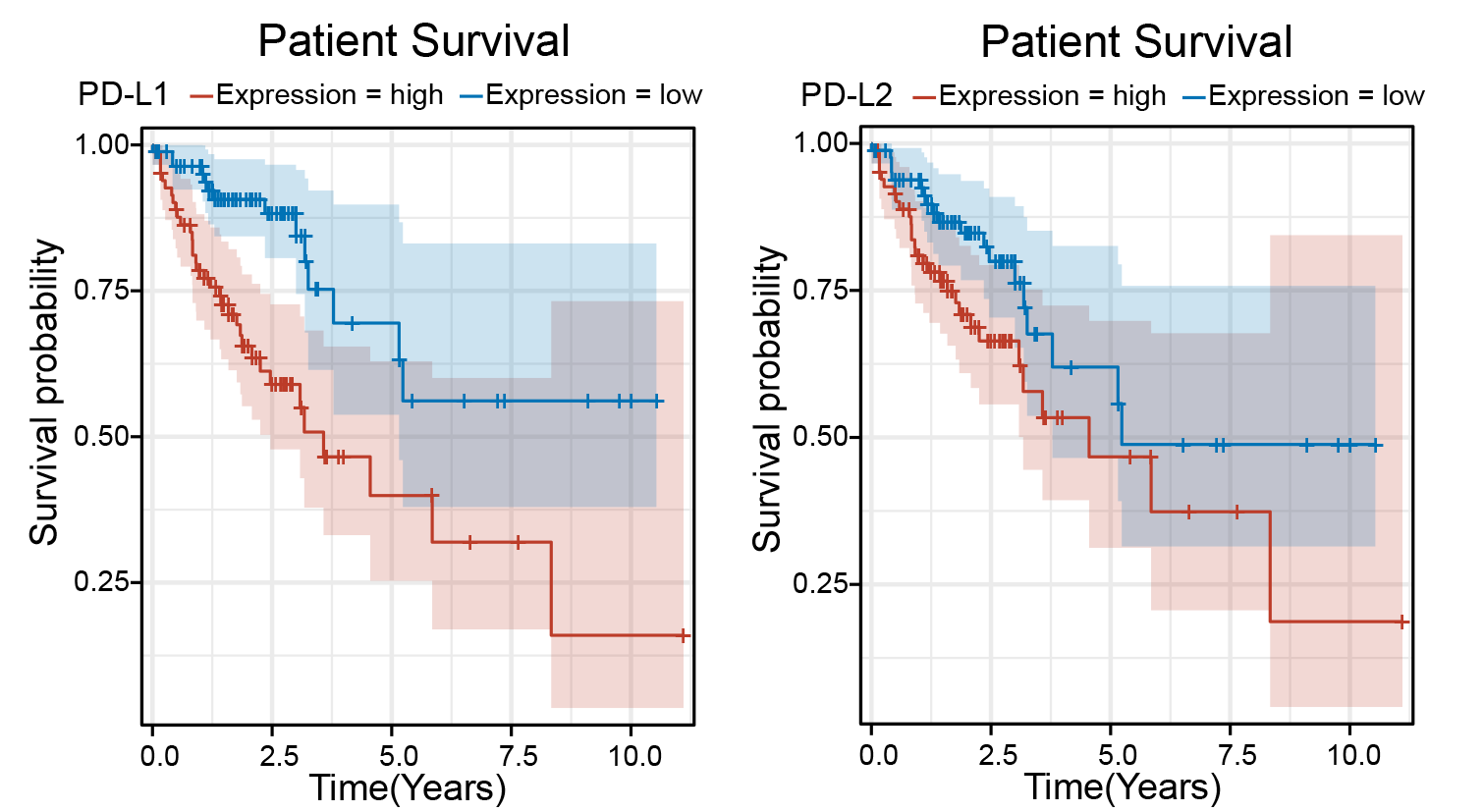

Supplement: Supplementary Figure 2 — Univariable Cox regression curves for overall survival of CRC patients, stratified by PD-L1 and PD-L2 expression levels. [file Image2.tif]
